# Dietary Risk Factors for Colon and Rectal Cancers: A Comparative Case-Control Study

**DOI:** 10.2188/jea.16.125

**Published:** 2006-05-19

**Authors:** Kenji Wakai, Kaoru Hirose, Keitaro Matsuo, Hidemi Ito, Kiyonori Kuriki, Takeshi Suzuki, Tomoyuki Kato, Takashi Hirai, Yukihide Kanemitsu, Kazuo Tajima

**Affiliations:** 1 Division of Epidemiology and Prevention, Aichi Cancer Center Research Institute.; 2 Department of Internal Medicine and Molecular Science, Nagoya City University Graduate School of Medical Science.; 3 Department of Gastroenterological Surgery, Aichi Cancer Center Hospital.

**Keywords:** Diet, Colonic Neoplasms, Rectal Neoplasms, Case-Control Studies, Japan

## Abstract

**BACKGROUND:**

In Japan, the incidence rate of colon cancer has more rapidly increased than that of rectal cancer. The differential secular trends may be due to different dietary factors in the development of colon and rectal cancers.

**METHODS:**

To compare dietary risk factors between colon and rectal cancers, we undertook a case-control study at Aichi Cancer Center Hospital, Japan. Subjects were 507 patients with newly diagnosed colon (n = 265) and rectal (n = 242) cancers, and 2,535 cancer-free outpatients (controls). Intakes of nutrients and food groups were assessed with a food frequency questionnaire, and multivariate-adjusted odds ratios (ORs) were estimated using unconditional logistic models.

**RESULTS:**

We found a decreasing risk of colon cancer with increasing intakes of calcium and insoluble dietary fiber; the multivariate ORs across quartiles of intake were 1.00, 0.90, 0.80, and 0.67 (trend p = 0.040), and 1.00, 0.69, 0.64, and 0.65 (trend p = 0.027), respectively. For rectal cancer, a higher consumption of carotene and meat was associated with a reduced risk; the corresponding ORs were 1.00, 1.10, 0.71, and 0.70 for carotene (trend p = 0.028), and 1.00, 0.99, 0.68, and 0.72 for meat (trend p = 0.036). Carbohydrate intake was positively correlated with the risk of rectal cancer (ORs over quartiles: 1.00, 1.14, 1.42, and 1.54; trend p = 0.048). This association was stronger in women, while fat consumption was inversely correlated with the risk of female colon and rectal cancers.

**CONCLUSIONS:**

Dietary risk factors appear to considerably differ between colon and rectal cancers.

In Japan, the age-standardized incidence rate of colorectal cancer increased until around 1990 and has leveled off thereafter.^1^ It is now at among the highest levels in the world; the incidence rate standardized with the World Population was estimated to be 49.9 (per 100,000 population) in men and 27.2 in women in 1999.^1^

The incidence of colon cancer has increased more rapidly than that of rectal cancer. Between 1975 and 1999, the colon-to-rectal ratio of incidence (standardized with the World Population) rose from 0.85 to 1.67 in men and from 1.17 to 2.13 in women.^1^^,^^2^ The ratio greatly varies among countries,^3^ being much higher in cancer registries in the United States and Canada (median = 2.1 in men and 2.6 in women) than those in Asian countries excluding Japan (1.2 in men and 1.4 in women). Registries in European countries have intermediate values (1.5 in men and 2.0 in women).

If dietary risk factors of colon cancer differ from those of rectal cancer, the different secular trends in incidence between the two sites and international variation in the colon-to-rectal ratio of incidence may partly be explained by changes and international variation in dietary habits. In Asian countries, however, only a small number of studies^4^^-^^7^ have examined differences in dietary risk factors between cancers of the colon and rectum. It is therefore not clear whether the predominant increase in incidence of colon cancer in Japan is ascribable to changes in diet. We need further data to know why the proportion of rectal cancer in all colorectal cancer cases is relatively high in Asian countries.

To further address these issues, we conducted the present case-control study comparing dietary risk factors between colon and rectal cancers in the Hospital-based Epidemiologic Research Program at Aichi Cancer Center (HERPACC).

## METHODS

### The Hospital-based Epidemiologic Research Program at Aichi Cancer Center

HERPACC was initiated in Aichi Cancer Center Hospital (ACCH), Nagoya, in 1988, with information on lifestyle factors collected from all first-visit outpatients, using a self-administered questionnaire checked by a trained interviewer. Each patient is asked about his or her lifestyle including dietary habits when healthy or before the current symptoms developed. The questionnaire data are loaded into the HERPACC database and routinely linked with the hospital cancer registry system to update the data on cancer incidence. Written informed consent for participation is obtained from each patient. The ethical board of Aichi Cancer Center reviewed and approved the protocol of this investigation. Further details of HERPACC have been described elsewhere.^6^^,^^8^

### Cases and Controls

The present study is based on data collected between January 2001 and September 2004 because the present version of the food frequency questionnaire was adopted in January 2001. Among all first-visit outpatients during this period (n = 25,941), the questionnaire was given to 21,417 (82.6%). Of the remaining 4,524 patients (17.4%), 2,041 (7.9%) were excluded because of the absence of an interviewer and so were 1,222 (4.7%) due to a consultation visit by someone other than patients themselves. Others were left out because they were too young (< 18 years) or too ill to fill out the form or for other miscellaneous reasons. Of the 21,417 outpatients who were asked to complete the questionnaire, 20,814 (97.2%) provided adequate responses to the questionnaire.

Patients aged 20 to 79 years with cancers of the colon (n = 323) or rectum (n = 276) (International Statistical Classification of Diseases and Related Health Problems, 10th Revision [ICD-10]: C18 and C20), newly and histopathologically diagnosed, were deemed to be potential cases. We excluded 85 patients with a prior history of cancer and seven with an implausibly high or low estimated intake of total energy (<500 or 3,500+ kcal/day), leaving 507 cases eligible for the analysis (colon cancer: n = 265; rectal cancer: n = 242).

We randomly selected five controls for each case from the 14,931 cancer-free individuals, with matching for age (5-year strata), sex, and calendar year of the first visit. Those with a history of cancer (n = 1,188) and an extreme value of energy intake (n = 125) were excluded as in the case patients. Finally, 2,535 controls were included in this study.

### Diet and Other Exposure Data

The HERPACC questionnaire applied included items on demographic characteristics, family and individual medical history, height and weight, exercise, smoking and drinking habits, and vitamin use, as well as consumption of selected foods and beverages.

The dietary component of the questionnaire comprised 47 food items.^9^ We asked the subjects about the average intake frequency without specifying portion size, during the period of one year before onset of the present disease or before the interview. For staple foods such as rice, bread, and noodles, the usual number of bowls or slices consumed at one time, as well as the intake frequency, was inquired for breakfast, lunch, and supper, separately. The frequency of alcohol consumption was asked with the usual amount on one occasion. Nutrient intakes and food group consumption were estimated assuming the standard portion sizes.

Energy-adjusted intakes of nutrients and food groups were calculated by the residual method,^10^ with natural logarithms used to improve the normality of their distribution except for the ratio of n-6 fatty acid intake to that of n-3 fatty acids. The food frequency questionnaire was validated by referring to 3-day weighed dietary records as a standard.^9^ The de-attenuated correlation coefficients for energy-adjusted intakes of nutrients for the present analysis ranged from 0.12 to 0.86 in men (median = 0.43) and from 0.17 to 0.64 in women (0.38). The coefficients (not de-attenuated) for energy-adjusted consumption of food groups varied from 0.19 to 0.57 in men (median = 0.42) and from 0.19 to 0.61 in women (0.42).

### Statistical Analysis

Body mass index (BMI) at baseline was calculated from reported height and weight: BMI = (weight in kg)/(height in m)^2^.

To assess the strength of the associations between intakes of nutrients or food groups and risk of colon or rectal cancer, odds ratios (ORs) were computed. To directly compare dietary risk factors between colon and rectal cancers based on a common control group, we pooled controls matched to colon cancer cases and those matched to rectal cancer cases in the analysis. Cases and controls were categorized into four groups according to sex-specific quartile levels of energy-adjusted intakes of nutrients or food groups among controls. The ORs with 95% confidence intervals (CIs) for the second, third, and highest quartiles versus the lowest were estimated using unconditional logistic models^11^ adjusted for the matching variables and potential confounding factors.^12^^-^^14^

The ORs were adjusted for sex, age (as a continuous variable), calendar year of the first visit to the hospital (2001, 2002, or 2003-2004), season of first visit (spring, summer, autumn, or winter), reason for the visit (self recommendation, recommendation by family or friends, referral by physicians, secondary screening, or others), family history of colorectal cancer in parents and/or siblings (yes or no), BMI (<20.0, 20.0-24.9, 25.0-29.9, or 30.0+ kg/m^2^), exercise (none, <0.50, 0.50-0.99, or 1.00+ hours/day), alcohol drinking (nondrinkers, ex-drinkers, or current drinkers who daily consumed <1.0, 1.0-1.9, or 2.0+ Japanese drinks [one Japanese drink is equivalent to 23g of ethanol]), smoking habit (nonsmokers, ex-smokers, or current smokers), multivitamin use (at least once per week for one year or longer; yes or no), and total energy intake (as a continuous variable). Missing values for each covariate were treated as an additional category in the variable and were included in the logistic model. As a basis for the trend tests, we assigned scores of 0, 1, 2, and 3 to the first (or lowest), second, third, and fourth quartiles of nutrient intakes or food group consumption, respectively, and included the score in the model. All p values were two-sided, and all the analyses were performed using the Statistical Analysis System^®^, release 8.2.^15^

## RESULTS

Table 1 shows the distribution of cases and controls by background characteristics; sex, age, and calendar year of the first visit were exactly matched between cases and controls. Values for mean age ± standard deviation were 61.7 ± 9.2 and 61.6 ± 9.3 years in cases and controls for colon cancer, respectively. The corresponding figures were 58.6 ± 10.7 and 58.5 ± 10.6 years for the rectal cancer subjects. As expected, the case group included a higher proportion of patients referred by physicians than the control group.

**Table 1.** Background characteristics of cases and controls for colon and rectal cancers.

	Colon cancer				Rectal cancer			
	
	Cases		Controls		Cases		Controls	
	(n = 265)		(n = 1,325)		(n = 242)		(n = 1,210)	
Sex								
Men	149	(56.2)	745	(56.2)	146	(60.3)	730	(60.3)
Women	116	(43.8)	580	(43.8)	96	(39.7)	480	(39.7)

Age (years)								
20-29	1	(0.4)	5	(0.4)	1	(0.4)	5	(0.4)
30-39	4	(1.5)	20	(1.5)	17	(7.0)	85	(7.0)
40-49	19	(7.2)	95	(7.2)	22	(9.1)	110	(9.1)
50-59	83	(31.3)	415	(31.3)	83	(34.3)	415	(34.3)
60-69	106	(40.0)	530	(40.0)	81	(33.5)	405	(33.5)
70-79	52	(19.6)	260	(19.6)	38	(15.7)	190	(15.7)

Calendar year of first visit								
2001	73	(27.5)	365	(27.5)	67	(27.7)	335	(27.7)
2002	83	(31.3)	415	(31.3)	69	(28.5)	345	(28.5)
2003-2004	109	(41.1)	545	(41.1)	106	(43.8)	530	(43.8)

Season of first visit to the hospital								
Spring	66	(24.9)	332	(25.1)	67	(27.7)	310	(25.6)
Summer	92	(34.7)	415	(31.3)	62	(25.6)	348	(28.8)
Autumn	51	(19.2)	341	(25.7)	62	(25.6)	314	(26.0)
Winter	56	(21.1)	237	(17.9)	51	(21.1)	238	(19.7)

Reason to visit the hospital								
Self recommendation	37	(14.0)	407	(30.7)	32	(13.2)	361	(29.8)
Recommendation by family or friends	49	(18.5)	289	(21.8)	47	(19.4)	245	(20.2)
Referral by physicians	136	(51.3)	366	(27.6)	126	(52.1)	301	(24.9)
Secondary screening	38	(14.3)	243	(18.3)	36	(14.9)	284	(23.5)
Others	5	(1.9)	20	(1.5)	1	(0.4)	19	(1.6)

Family history of colorectal cancer in parents and/or siblings								
Yes	38	(14.3)	100	(7.5)	24	(9.9)	77	(6.4)
No	227	(85.7)	1,225	(92.5)	218	(90.1)	1,133	(93.6)

Body mass index (kg/m^2^)								
< 20.0	40	(15.2)	226	(17.3)	38	(15.8)	206	(17.1)
20.0-24.9	166	(62.9)	791	(60.4)	146	(60.8)	748	(62.2)
25.0-29.9	54	(20.5)	276	(21.1)	50	(20.8)	229	(19.0)
≥ 30.0	4	(1.5)	16	(1.2)	6	(2.5)	20	(1.7)

Exercise (hours/day)								
None	75	(29.1)	337	(26.0)	67	(29.1)	330	(27.8)
< 0.50	103	(39.9)	564	(43.5)	93	(40.4)	513	(43.3)
0.50-0.99	49	(19.0)	229	(17.7)	47	(20.4)	184	(15.5)
≥ 1.00	31	(12.0)	167	(12.9)	23	(10.0)	158	(13.3)

Alcohol drinking								
Nondrinkers	130	(49.4)	627	(47.8)	99	(41.8)	508	(42.6)
Ex-drinkers	25	(9.5)	103	(7.9)	16	(6.8)	82	(6.9)
Current drinkers (Japanese drinks/day)								
< 1.0	65	(24.7)	309	(23.6)	60	(25.3)	338	(28.4)
1.0-1.9	22	(8.4)	134	(10.2)	32	(13.5)	130	(10.9)
≥ 2.0	21	(8.0)	139	(10.6)	30	(12.7)	134	(11.2)

Smoking								
Nonsmokers	132	(49.8)	667	(50.4)	104	(43.0)	560	(46.4)
Ex-smokers	78	(29.4)	379	(28.6)	65	(26.9)	342	(28.3)
Current smokers	55	(20.8)	277	(20.9)	73	(30.2)	306	(25.3)

Multivitamin use (at least once per week for one year or longer)								
Yes	24	(9.1)	113	(8.5)	15	(6.2)	99	(8.2)
No	241	(90.9)	1,212	(91.5)	227	(93.8)	1,111	(91.8)
Energy intake (kcal/day, mean ± SD)	1,580 ± 351		1,616 ± 342		1,609 ± 370		1,634 ± 352	

Percentages in parentheses

Cases of both colon and rectal cancers were more likely to have a family history of colorectal cancer than the controls. Other characteristics, such as season of first visit to the hospital, BMI, exercise, drinking and smoking habits, multivitamin use, and energy intake, were similarly distributed in cases and controls.

The greater the intake of calcium and insoluble dietary fiber, the lower the multivariate OR (OR2) for colon cancer (Table 2; trend p = 0.040 for calcium and 0.027 for insoluble dietary fiber). The risks for the highest quartile of intake of calcium and insoluble dietary fiber were 33% and 35% lower than those for the lowest quartile, respectively (OR2: 0.67 [95% CI: 0.46-1.00] for calcium and 0.65 [95% CI: 0.45-0.96] for insoluble dietary fiber). Inverse associations were also suggested between colon cancer risk and intakes of protein, fat, vitamin C, and total dietary fiber (trend p for OR2 < 0.10).

**Table 2.** Odds ratios (ORs) for colon cancer by quartile (Q1-Q4) of energy-adjusted intake of nutrients or food groups in men and women (265 cases and 2,535 controls).

Nutrients/food groups	OR1 (95% confidence interval)*					OR2 (95% confidence interval)^†^				
	
	Q1	Q2	Q3	Q4	Trend p	Q1	Q2	Q3	Q4	Trend p
Protein	1.00	0.82 (0.58 - 1.17)	0.76 (0.53 - 1.08)	0.84 (0.60 - 1.19)	0.28	1.00	0.74 (0.52 -1.07)	0.65 (0.45 - 0.95)	0.74 (0.51 - 1.07)	0.084
Fat	1.00	1.06 (0.76 - 1.49)	0.72 (0.50 - 1.05)	0.89 (0.63 - 1.28)	0.22	1.00	1.00 (0.71 - 1.42)	0.67 (0.45 - 0.98)	0.78 (0.54 - 1.13)	0.064
Carbohydrate	1.00	1.14 (0.79 - 1.66)	1.09 (0.75 - 1.59)	1.33 (0.93 - 1.91)	0.16	1.00	1.15 (0.77 - 1.70)	1.02 (0.67 - 1.53)	1.16 (0.76 - 1.79)	0.65
Calcium	1.00	1.03 (0.73 - 1.46)	0.90 (0.63 - 1.29)	0.78 (0.54 - 1.13)	0.14	1.00	0.90 (0.62 - 1.28)	0.80 (0.55 - 1.17)	0.67 (0.46 - 1.00)	0.040
Carotene	1.00	0.78 (0.54 - 1.13)	1.00 (0.70 - 1.42)	0.96 (0.67 - 1.37)	0.85	1.00	0.75 (0.51 - 1.10)	0.89 (0.61 - 1.29)	0.87 (0.59 - 1.28)	0.69
Retinol	1.00	0.87 (0.60 - 1.26)	0.93 (0.65 - 1.34)	1.12 (0.79 - 1.59)	0.44	1.00	0.86 (0.58 - 1.25)	1.00 (0.69 - 1.46)	1.06 (0.73 - 1.54)	0.57
Vitamin D	1.00	1.03 (0.72 - 1.47)	0.88 (0.61 - 1.28)	1.02 (0.71 - 1.45)	0.88	1.00	1.03 (0.71 - 1.49)	0.85 (0.58 - 1.24)	1.04 (0.72 - 1.51)	0.92
Vitamin E	1.00	0.81 (0.56 - 1.16)	1.03 (0.73 - 1.45)	0.79 (0.55 - 1.14)	0.44	1.00	0.75 (0.52 - 1.09)	0.95 (0.67 - 1.36)	0.73 (0.50 - 1.08)	0.28
Folate	1.00	0.73 (0.51 - 1.05)	0.89 (0.62 - 1.26)	0.88 (0.62 - 1.25)	0.70	1.00	0.65 (0.44 - 0.95)	0.82 (0.57 - 1.19)	0.75 (0.51 - 1.11)	0.32
Vitamin C	1.00	0.78 (0.54 - 1.12)	0.94 (0.66 - 1.33)	0.79 (0.55 - 1.14)	0.38	1.00	0.72 (0.49 - 1.04)	0.84 (0.58 - 1.21)	0.65 (0.44 - 0.96)	0.072
SFA^‡^	1.00	0.89 (0.62 - 1.28)	0.87 (0.60 - 1.24)	1.01 (0.71 - 1.43)	1.00	1.00	0.77 (0.53 - 1.12)	0.77 (0.53 - 1.12)	0.83 (0.57 - 1.20)	0.35
MUFA^§^	1.00	1.02 (0.71 - 1.46)	1.09 (0.77 - 1.56)	1.04 (0.72 - 1.51)	0.73	1.00	0.94 (0.65 - 1.36)	1.04 (0.71 - 1.52)	0.92 (0.62 - 1.36)	0.80
PUFA^∥^	1.00	0.92 (0.64 - 1.32)	1.03 (0.72 - 1.46)	1.01 (0.71 - 1.45)	0.80	1.00	0.79 (0.54 - 1.15)	1.00 (0.69 - 1.45)	0.90 (0.61 - 1.31)	0.88
Cholesterol	1.00	0.86 (0.60 - 1.23)	0.95 (0.67 - 1.36)	0.92 (0.64 - 1.30)	0.76	1.00	0.81 (0.56 - 1.17)	0.88 (0.61 - 1.26)	0.77 (0.52 - 1.13)	0.25
Soluble dietary fiber	1.00	0.90 (0.64 - 1.28)	0.83 (0.58 - 1.18)	0.78 (0.55 - 1.13)	0.16	1.00	0.89 (0.62 - 1.28)	0.77 (0.53 - 1.12)	0.75 (0.52 - 1.10)	0.11
Insoluble dietary fiber	1.00	0.74 (0.52 - 1.05)	0.74 (0.52 - 1.05)	0.72 (0.51 - 1.03)	0.078	1.00	0.69 (0.48 - 1.00)	0.64 (0.44 - 0.93)	0.65 (0.45 - 0.96)	0.027
Total dietary fiber	1.00	0.84 (0.59 - 1.20)	0.79 (0.55 - 1.13)	0.76 (0.53 - 1.09)	0.13	1.00	0.78 (0.54 - 1.12)	0.71 (0.49 - 1.03)	0.72 (0.49 - 1.05)	0.074
n-3 PUFA	1.00	0.91 (0.63 - 1.30)	1.05 (0.74 - 1.49)	0.95 (0.66 - 1.36)	0.98	1.00	0.90 (0.62 - 1.30)	1.02 (0.71 - 1.47)	0.89 (0.61 - 1.30)	0.72
n-6 PUFA	1.00	0.88 (0.61 - 1.26)	1.10 (0.77 - 1.56)	1.02 (0.71 - 1.45)	0.65	1.00	0.75 (0.51 - 1.09)	1.01 (0.70 - 1.46)	0.84 (0.57 - 1.24)	0.77
n-6 PUFA/n-3 PUFA	1.00	0.93 (0.65 - 1.34)	1.22 (0.87 - 1.72)	0.90 (0.62 - 1.30)	0.97	1.00	0.95 (0.65 - 1.38)	1.24 (0.87 - 1.77)	0.84 (0.57 - 1.23)	0.71

Soy foods	1.00	1.37 (0.96 - 1.95)	0.97 (0.66 - 1.42)	1.07 (0.74 - 1.55)	0.76	1.00	1.41 (0.98 - 2.04)	0.99 (0.67 - 1.47)	1.02 (0.69 - 1.50)	0.59
Meat	1.00	1.06 (0.74 - 1.52)	1.17 (0.82 - 1.68)	1.06 (0.74 - 1.54)	0.62	1.00	1.11 (0.76 - 1.61)	1.19 (0.82 - 1.73)	0.95 (0.65 - 1.41)	0.93
Fish	1.00	1.20 (0.84 - 1.72)	1.03 (0.71 - 1.50)	1.11 (0.77 - 1.60)	0.78	1.00	1.18 (0.81 - 1.70)	1.00 (0.68 - 1.47)	1.10 (0.75 - 1.62)	0.83
Green-yellow vegetables	1.00	0.89 (0.63 - 1.27)	0.88 (0.62 - 1.25)	0.79 (0.55 - 1.14)	0.23	1.00	0.86 (0.59 - 1.23)	0.80 (0.55 - 1.16)	0.75 (0.51 - 1.10)	0.13
Other vegetables	1.00	1.17 (0.83 - 1.67)	0.83 (0.57 - 1.21)	1.09 (0.76 - 1.56)	0.90	1.00	1.11 (0.77 - 1.59)	0.78 (0.52 - 1.15)	0.96 (0.66 - 1.40)	0.45
Fruit	1.00	0.80 (0.56 - 1.15)	0.74 (0.51 - 1.06)	0.90 (0.64 - 1.28)	0.50	1.00	0.73 (0.50 - 1.06)	0.71 (0.48 - 1.04)	0.73 (0.50 - 1.06)	0.12

* : adjusted for sex, age, and year of first visit.

† : further adjusted for season of first visit to the hospital, reason for the visit, family history of colorectal cancer, body mass index, exercise, alcohol drinking, smoking, multivitamin use, and energy intake.

‡ : saturated fatty acids

§ : monounsaturated fatty acids

∥ : polyunsaturated fatty acids

We found a decreased risk of rectal cancer associated with higher intakes of carotene and meat (Table 3; trend p for OR2 = 0.028 for carotene and 0.036 for meat). A negative correlation was also suggested between the risk of rectal cancer and intake of vitamin E (trend p for OR2 = 0.072). On the other hand, an increasing risk was found with increasing intake of carbohydrate (trend p for OR2 = 0.048).

**Table 3.** Odds ratios (ORs) for rectal cancer by quartile (Q1-Q4) of energy-adjusted intake of nutrients or food groups in men and women (242 cases and 2,535 controls).

Nutrients/food groups	OR1 (95% confidence interval)*					OR2 (95% confidence interval)^†^				
	
	Q1	Q2	Q3	Q4	Trend p	Q1	Q2	Q3	Q4	Trend p
Protein	1.00	0.81 (0.57 - 1.17)	0.92 (0.64 - 1.31)	0.70 (0.48 - 1.03)	0.13	1.00	0.83 (0.57 - 1.22)	0.91 (0.62 - 1.32)	0.71 (0.47 - 1.06)	0.16
Fat	1.00	0.86 (0.60 - 1.24)	0.85 (0.59 - 1.23)	0.73 (0.50 - 1.07)	0.12	1.00	0.86 (0.59 - 1.25)	0.90 (0.61 - 1.31)	0.73 (0.49 - 1.09)	0.17
Carbohydrate	1.00	1.03 (0.70 - 1.53)	1.27 (0.87 - 1.86)	1.37 (0.94 - 2.00)	0.060	1.00	1.14 (0.75 - 1.74)	1.42 (0.93 - 2.19)	1.54 (0.96 - 2.47)	0.048
Calcium	1.00	1.34 (0.92 - 1.94)	1.30 (0.89 - 1.89)	0.93 (0.62 - 1.40)	0.76	1.00	1.24 (0.85 - 1.82)	1.33 (0.89 - 1.97)	0.97 (0.63 - 1.50)	0.99
Carotene	1.00	1.07 (0.76 - 1.52)	0.75 (0.52 - 1.10)	0.74 (0.50 - 1.09)	0.044	1.00	1.10 (0.77 - 1.59)	0.71 (0.47 - 1.06)	0.70 (0.46 - 1.08)	0.028
Retinol	1.00	1.07 (0.74 - 1.55)	1.03 (0.71 - 1.50)	0.93 (0.63 - 1.37)	0.69	1.00	1.09 (0.74 - 1.60)	1.10 (0.75 - 1.62)	0.92 (0.61 - 1.39)	0.75
Vitamin D	1.00	0.81 (0.56 - 1.18)	0.88 (0.61 - 1.27)	0.95 (0.66 - 1.38)	0.88	1.00	0.77 (0.53 - 1.13)	0.81 (0.55 - 1.20)	0.97 (0.66 - 1.44)	0.91
Vitamin E	1.00	0.68 (0.47 - 0.99)	0.80 (0.56 - 1.13)	0.65 (0.45 - 0.94)	0.047	1.00	0.65 (0.44 - 0.95)	0.78 (0.54 - 1.13)	0.65 (0.43 - 0.97)	0.072
Folate	1.00	1.00 (0.70 - 1.42)	0.78 (0.53 - 1.14)	0.83 (0.57 - 1.21)	0.19	1.00	1.00 (0.69 - 1.45)	0.80 (0.53 - 1.19)	0.81 (0.53 - 1.23)	0.20
Vitamin C	1.00	0.91 (0.64 - 1.31)	0.78 (0.54 - 1.13)	0.84 (0.57 - 1.22)	0.24	1.00	0.94 (0.65 - 1.36)	0.82 (0.56 - 1.22)	0.84 (0.55 - 1.26)	0.31
SFA^‡^	1.00	1.25 (0.86 - 1.81)	1.21 (0.83 - 1.76)	0.89 (0.60 - 1.33)	0.58	1.00	1.19 (0.81 - 1.75)	1.25 (0.84 - 1.85)	0.86 (0.56 - 1.33)	0.57
MUFA^§^	1.00	0.76 (0.52 - 1.09)	0.66 (0.45 - 0.96)	0.82 (0.57 - 1.18)	0.22	1.00	0.73 (0.50 - 1.08)	0.64 (0.43 - 0.96)	0.76 (0.51 - 1.14)	0.15
PUFA^∥^	1.00	0.66 (0.45 - 0.96)	0.89 (0.62 - 1.27)	0.80 (0.56 - 1.16)	0.49	1.00	0.59 (0.40 - 0.88)	0.90 (0.62 - 1.30)	0.76 (0.51 - 1.12)	0.47
Cholesterol	1.00	1.04 (0.72 - 1.49)	0.79 (0.54 - 1.17)	0.97 (0.67 - 1.41)	0.57	1.00	1.06 (0.72 - 1.54)	0.79 (0.53 - 1.19)	0.89 (0.59 - 1.33)	0.33
Soluble dietary fiber	1.00	0.97 (0.68 - 1.39)	1.02 (0.71 - 1.46)	0.71 (0.48 - 1.06)	0.16	1.00	0.99 (0.68 - 1.43)	1.06 (0.73 - 1.54)	0.74 (0.49 - 1.12)	0.25
Insoluble dietary fiber	1.00	1.04 (0.73 - 1.49)	1.08 (0.75 - 1.55)	0.75 (0.50 - 1.12)	0.24	1.00	1.03 (0.70 - 1.49)	1.07 (0.73 - 1.56)	0.78 (0.51 - 1.20)	0.35
Total dietary fiber	1.00	0.91 (0.63 - 1.31)	1.01 (0.71 - 1.45)	0.72 (0.49 - 1.08)	0.21	1.00	0.88 (0.60 - 1.28)	1.01 (0.70 - 1.47)	0.76 (0.50 - 1.15)	0.35
n-3 PUFA	1.00	0.93 (0.65 - 1.34)	0.83 (0.57 - 1.20)	0.86 (0.59 - 1.24)	0.33	1.00	0.92 (0.63 - 1.34)	0.83 (0.56 - 1.23)	0.85 (0.57 - 1.27)	0.37
n-6 PUFA	1.00	0.99 (0.68 - 1.44)	0.87 (0.59 - 1.27)	1.01 (0.70 - 1.47)	0.90	1.00	0.93 (0.63 - 1.38)	0.85 (0.57 - 1.27)	0.97 (0.65 - 1.45)	0.78
n-6 PUFA/n-3 PUFA	1.00	0.87 (0.59 - 1.29)	1.03 (0.71 - 1.51)	1.23 (0.85 - 1.77)	0.18	1.00	0.88 (0.59 - 1.32)	1.03 (0.70 - 1.53)	1.23 (0.84 - 1.80)	0.21

Soy foods	1.00	1.11 (0.78 - 1.59)	0.81 (0.55 - 1.20)	1.03 (0.71 - 1.50)	0.73	1.00	1.19 (0.82 - 1.73)	0.85 (0.56 - 1.27)	1.03 (0.69 - 1.53)	0.70
Meat	1.00	0.93 (0.66 - 1.33)	0.65 (0.44 - 0.95)	0.76 (0.52 - 1.10)	0.050	1.00	0.99 (0.68 - 1.42)	0.68 (0.46 - 1.02)	0.72 (0.48 - 1.07)	0.036
Fish	1.00	0.77 (0.53 - 1.12)	0.81 (0.56 - 1.18)	1.00 (0.69 - 1.43)	0.99	1.00	0.75 (0.51 - 1.10)	0.75 (0.51 - 1.11)	1.03 (0.70 - 1.51)	0.98
Green-yellow vegetables	1.00	0.91 (0.64 - 1.30)	0.78 (0.54 - 1.14)	0.78 (0.54 - 1.14)	0.14	1.00	0.94 (0.65 - 1.35)	0.76 (0.52 - 1.13)	0.83 (0.55 - 1.24)	0.22
Other vegetables	1.00	1.19 (0.83 - 1.70)	0.90 (0.61 - 1.32)	0.84 (0.57 - 1.25)	0.21	1.00	1.13 (0.78 - 1.63)	0.92 (0.62 - 1.37)	0.78 (0.52 - 1.18)	0.16
Fruit	1.00	1.27 (0.88 - 1.84)	1.03 (0.69 - 1.52)	1.21 (0.82 - 1.78)	0.58	1.00	1.35 (0.92 - 1.97)	1.16 (0.77 - 1.76)	1.23 (0.81 - 1.87)	0.51

* : adjusted for sex, age, and year of first visit.

† : further adjusted for season of first visit to the hospital, reason for the visit, family history of colorectal cancer, body mass index, exercise, alcohol drinking, smoking, multivitamin use, and energy intake.

‡ : saturated fatty acids

§ : monounsaturated fatty acids

∥ : polyunsaturated fatty acids

In women, intakes of protein, fat, calcium, vitamin E, cholesterol, and total dietary fiber were inversely correlated with the risk of colon cancer (trend p < 0.10, Table 4), while no significant associations were noted in men. A reduced risk of rectal cancer associated with a higher consumption of carotene and meat was observed particularly in women (Table 5). Inverse associations were also found for fat, vitamin E, folate, monounsaturated and n-6 polyunsaturated fatty acids, and green-yellow vegetables in women (trend p < 0.10). In contrast, women who took diet high in carbohydrate were at more than twice the risk of developing rectal cancer. The ORs for the third and the highest quartiles were 2.14 (95% CI: 1.05-4.36) and 2.53 (95% CI: 1.22-5.24; trend p = 0.003), respectively. An increasing risk with an increasing ratio of dietary n-6 polyunsaturated fatty acids (PUFA) to n-3 PUFA was detected for male rectal cancer (trend p = 0.042).

**Table 4.** Odds ratios* (ORs) for colon cancer according to quartiles (Q1-Q4) of energy-adjusted intake of nutrients or food groups by sex.

Nutrients/food groups	Men (149 cases and 1,475 controls)					Women (116 cases and 1,060 controls)				
	
	Q1	Q2	Q3	Q4	Trend p	Q1	Q2	Q3	Q4	Trend p
Protein	1.00	0.79 (0.48 - 1.31)	0.79 (0.48 - 1.30)	0.78 (0.47 - 1.31)	0.38	1.00	0.71 (0.41 - 1.24)	0.48 (0.26 - 0.87)	0.67 (0.38 - 1.18)	0.077
Fat	1.00	1.05 (0.65 - 1.69)	0.70 (0.41 - 1.19)	0.94 (0.57 - 1.55)	0.49	1.00	0.96 (0.56 - 1.64)	0.60 (0.33 - 1.09)	0.59 (0.32 - 1.07)	0.035
Carbohydrate	1.00	1.21 (0.71 - 2.05)	0.89 (0.51 - 1.57)	0.81 (0.43 - 1.53)	0.33	1.00	0.93 (0.50 - 1.74)	1.04 (0.55 - 1.95)	1.51 (0.82 - 2.78)	0.15
Calcium	1.00	0.89 (0.54 - 1.47)	0.85 (0.51 - 1.41)	0.77 (0.46 - 1.30)	0.33	1.00	0.87 (0.51 - 1.51)	0.75 (0.42 - 1.33)	0.52 (0.28 - 0.97)	0.035
Carotene	1.00	0.71 (0.42 - 1.21)	0.95 (0.57 - 1.57)	0.86 (0.50 - 1.49)	0.88	1.00	0.81 (0.45 - 1.46)	0.81 (0.46 - 1.44)	0.92 (0.51 - 1.65)	0.78
Retinol	1.00	0.74 (0.44 - 1.24)	0.80 (0.48 - 1.33)	0.99 (0.60 - 1.63)	0.94	1.00	1.04 (0.57 - 1.88)	1.34 (0.74 - 2.40)	1.20 (0.67 - 2.17)	0.40
Vitamin D	1.00	0.98 (0.59 - 1.61)	1.02 (0.63 - 1.67)	0.97 (0.59 - 1.61)	0.96	1.00	1.09 (0.62 - 1.91)	0.63 (0.34 - 1.18)	1.19 (0.67 - 2.11)	0.96
Vitamin E	1.00	0.82 (0.49 - 1.37)	1.03 (0.63 - 1.68)	1.02 (0.61 - 1.71)	0.71	1.00	0.72 (0.41 - 1.24)	0.93 (0.55 - 1.59)	0.48 (0.25 - 0.90)	0.069
Folate	1.00	0.88 (0.53 - 1.45)	0.75 (0.45 - 1.27)	0.87 (0.51 - 1.48)	0.52	1.00	0.48 (0.26 - 0.89)	0.95 (0.55 - 1.63)	0.68 (0.38 - 1.23)	0.56
Vitamin C	1.00	0.74 (0.45 - 1.23)	0.74 (0.45 - 1.24)	0.72 (0.42 - 1.22)	0.26	1.00	0.74 (0.41 - 1.32)	1.03 (0.59 - 1.78)	0.61 (0.33 - 1.12)	0.25
SFA^†^	1.00	0.88 (0.53 - 1.46)	0.87 (0.52 - 1.46)	0.95 (0.57 - 1.60)	0.89	1.00	0.64 (0.36 - 1.14)	0.62 (0.35 - 1.10)	0.66 (0.38 - 1.17)	0.16
MUFA^‡^	1.00	0.87 (0.51 - 1.47)	1.27 (0.76 - 2.11)	1.21 (0.70 - 2.09)	0.27	1.00	1.07 (0.62 - 1.83)	0.87 (0.48 - 1.56)	0.67 (0.36 - 1.24)	0.16
PUFA^§^	1.00	1.14 (0.68 - 1.92)	1.22 (0.72 - 2.06)	1.32 (0.78 - 2.26)	0.29	1.00	0.55 (0.31 - 0.99)	0.88 (0.51 - 1.52)	0.58 (0.32 - 1.03)	0.18
Cholesterol	1.00	1.19 (0.71 - 2.01)	1.24 (0.74 - 2.09)	1.02 (0.58 - 1.80)	0.93	1.00	0.49 (0.28 - 0.87)	0.55 (0.31 - 0.95)	0.54 (0.31 - 0.95)	0.037
Soluble dietary fiber	1.00	0.95 (0.58 - 1.57)	0.91 (0.55 - 1.49)	0.77 (0.46 - 1.30)	0.32	1.00	0.85 (0.49 - 1.46)	0.61 (0.34 - 1.10)	0.77 (0.43 - 1.37)	0.22
Insoluble dietary fiber	1.00	0.62 (0.37 - 1.04)	0.72 (0.44 - 1.18)	0.67 (0.40 - 1.12)	0.20	1.00	0.84 (0.48 - 1.44)	0.58 (0.33 - 1.04)	0.69 (0.39 - 1.24)	0.11
Total dietary fiber	1.00	0.94 (0.57 - 1.56)	0.89 (0.53 - 1.47)	0.84 (0.50 - 1.43)	0.50	1.00	0.67 (0.38 - 1.17)	0.55 (0.31 - 0.97)	0.63 (0.35 - 1.13)	0.073
n-3 PUFA	1.00	1.03 (0.62 - 1.71)	1.14 (0.69 - 1.89)	0.97 (0.57 - 1.66)	0.98	1.00	0.72 (0.40 - 1.28)	0.88 (0.50 - 1.54)	0.81 (0.45 - 1.44)	0.60
n-6 PUFA	1.00	0.83 (0.48 - 1.43)	1.29 (0.78 - 2.13)	1.09 (0.63 - 1.87)	0.40	1.00	0.71 (0.41 - 1.24)	0.82 (0.46 - 1.46)	0.63 (0.35 - 1.12)	0.17
n-6 PUFA/n-3 PUFA	1.00	0.96 (0.58 - 1.59)	1.29 (0.80 - 2.07)	0.79 (0.48 - 1.31)	0.63	1.00	0.91 (0.51 - 1.64)	1.19 (0.68 - 2.07)	0.86 (0.47 - 1.57)	0.89

Soy foods	1.00	1.37 (0.83 - 2.26)	1.11 (0.66 - 1.88)	1.17 (0.69 - 1.96)	0.79	1.00	1.54 (0.88 - 2.69)	0.77 (0.42 - 1.43)	0.83 (0.44 - 1.54)	0.18
Meat	1.00	1.25 (0.75 - 2.09)	1.32 (0.79 - 2.20)	1.15 (0.68 - 1.95)	0.59	1.00	0.96 (0.54 - 1.69)	1.03 (0.58 - 1.83)	0.73 (0.40 - 1.34)	0.39
Fish	1.00	1.36 (0.82 - 2.26)	1.33 (0.79 - 2.21)	1.13 (0.66 - 1.92)	0.72	1.00	0.97 (0.55 - 1.70)	0.70 (0.38 - 1.29)	1.11 (0.63 - 1.95)	0.98
Green-yellow vegetables	1.00	0.97 (0.59 - 1.60)	0.96 (0.58 - 1.58)	0.88 (0.52 - 1.48)	0.62	1.00	0.90 (0.51 - 1.58)	0.71 (0.40 - 1.26)	0.69 (0.38 - 1.23)	0.15
Other vegetables	1.00	1.10 (0.68 - 1.80)	0.75 (0.44 - 1.28)	0.95 (0.58 - 1.58)	0.54	1.00	1.29 (0.74 - 2.27)	0.89 (0.48 - 1.62)	1.08 (0.60 - 1.94)	0.89
Fruit	1.00	0.57 (0.34 - 0.95)	0.67 (0.41 - 1.10)	0.67 (0.41 - 1.11)	0.19	1.00	1.00 (0.57 - 1.77)	0.75 (0.41 - 1.38)	0.90 (0.50 - 1.62)	0.54

* : adjusted for age, year of first visit to the hospital, season of first visit, reason for the visit, family history of colorectal cancer, body mass index, exercise, alcohol drinking, smoking, multivitamin use, and energy intake.

† : saturated fatty acids

‡ : monounsaturated fatty acids

§ : polyunsaturated fatty acids

95% confidence intervals in parentheses

**Table 5.** Odds ratios* (ORs) for rectal cancer according to quartiles (Q1-Q4) of energy-adjusted intake of nutrients or food groups by sex.

Nutrients/food groups	Men (146 cases and 1,475 controls)					Women (96 cases and 1,060 controls)				
	
	Q1	Q2	Q3	Q4	Trend p	Q1	Q2	Q3	Q4	Trend p
Protein	1.00	1.02 (0.61 - 1.69)	1.23 (0.75 - 2.01)	0.77 (0.43 - 1.37)	0.62	1.00	0.65 (0.35 - 1.21)	0.64 (0.35 - 1.20)	0.66 (0.36 - 1.24)	0.19
Fat	1.00	1.12 (0.67 - 1.88)	1.24 (0.74 - 2.09)	1.15 (0.67 - 1.96)	0.56	1.00	0.57 (0.31 - 1.02)	0.61 (0.33 - 1.12)	0.38 (0.19 - 0.75)	0.008
Carbohydrate	1.00	1.13 (0.67 - 1.92)	1.12 (0.63 - 2.00)	1.07 (0.54 - 2.10)	0.86	1.00	1.17 (0.55 - 2.52)	2.14 (1.05 - 4.36)	2.53 (1.22 - 5.24)	0.003
Calcium	1.00	1.44 (0.87 - 2.41)	1.40 (0.82 - 2.41)	1.26 (0.71 - 2.25)	0.51	1.00	1.11 (0.60 - 2.05)	1.27 (0.68 - 2.36)	0.73 (0.36 - 1.47)	0.53
Carotene	1.00	1.44 (0.87 - 2.36)	0.83 (0.47 - 1.45)	1.05 (0.58 - 1.91)	0.58	1.00	0.83 (0.46 - 1.48)	0.66 (0.36 - 1.23)	0.48 (0.24 - 0.95)	0.028
Retinol	1.00	1.12 (0.66 - 1.89)	1.39 (0.83 - 2.33)	1.18 (0.67 - 2.08)	0.40	1.00	1.09 (0.61 - 1.95)	0.82 (0.44 - 1.53)	0.76 (0.40 - 1.48)	0.31
Vitamin D	1.00	0.92 (0.56 - 1.51)	0.93 (0.56 - 1.53)	0.91 (0.54 - 1.54)	0.74	1.00	0.61 (0.32 - 1.16)	0.72 (0.38 - 1.37)	1.11 (0.60 - 2.03)	0.71
Vitamin E	1.00	0.87 (0.51 - 1.47)	1.18 (0.72 - 1.94)	1.00 (0.58 - 1.73)	0.69	1.00	0.48 (0.26 - 0.85)	0.47 (0.25 - 0.87)	0.42 (0.22 - 0.80)	0.006
Folate	1.00	1.17 (0.71 - 1.95)	1.06 (0.62 - 1.81)	1.14 (0.64 - 2.02)	0.77	1.00	0.87 (0.49 - 1.53)	0.60 (0.31 - 1.14)	0.59 (0.31 - 1.14)	0.063
Vitamin C	1.00	0.87 (0.53 - 1.41)	0.91 (0.55 - 1.52)	0.73 (0.42 - 1.27)	0.33	1.00	1.06 (0.58 - 1.94)	0.72 (0.37 - 1.41)	1.04 (0.55 - 1.97)	0.83
SFA^†^	1.00	1.14 (0.68 - 1.91)	1.32 (0.78 - 2.22)	1.02 (0.58 - 1.80)	0.83	1.00	1.39 (0.76 - 2.57)	1.21 (0.64 - 2.30)	0.72 (0.36 - 1.45)	0.32
MUFA^‡^	1.00	0.93 (0.55 - 1.57)	0.86 (0.50 - 1.48)	1.23 (0.71 - 2.14)	0.51	1.00	0.57 (0.31 - 1.04)	0.46 (0.24 - 0.88)	0.47 (0.24 - 0.89)	0.014
PUFA^§^	1.00	0.69 (0.40 - 1.19)	1.08 (0.65 - 1.79)	1.06 (0.63 - 1.78)	0.46	1.00	0.54 (0.29 - 1.01)	0.76 (0.43 - 1.36)	0.53 (0.27 - 1.01)	0.11
Cholesterol	1.00	1.15 (0.69 - 1.92)	1.05 (0.62 - 1.78)	1.11 (0.63 - 1.96)	0.82	1.00	0.98 (0.55 - 1.76)	0.54 (0.27 - 1.06)	0.73 (0.39 - 1.37)	0.15
Soluble dietary fiber	1.00	1.45 (0.88 - 2.38)	1.33 (0.80 - 2.23)	0.93 (0.53 - 1.65)	0.77	1.00	0.59 (0.32 - 1.09)	0.91 (0.51 - 1.63)	0.66 (0.34 - 1.26)	0.37
Insoluble dietary fiber	1.00	1.19 (0.72 - 1.98)	1.40 (0.85 - 2.33)	0.83 (0.46 - 1.50)	0.78	1.00	0.88 (0.49 - 1.58)	0.77 (0.41 - 1.42)	0.84 (0.44 - 1.60)	0.49
Total dietary fiber	1.00	1.04 (0.63 - 1.70)	1.17 (0.71 - 1.94)	0.83 (0.47 - 1.45)	0.68	1.00	0.69 (0.37 - 1.29)	0.92 (0.51 - 1.67)	0.78 (0.40 - 1.51)	0.64
n-3 PUFA	1.00	0.94 (0.56 - 1.57)	1.02 (0.61 - 1.69)	0.96 (0.56 - 1.65)	0.97	1.00	1.02 (0.57 - 1.82)	0.65 (0.34 - 1.25)	0.80 (0.42 - 1.53)	0.29
n-6 PUFA	1.00	1.07 (0.63 - 1.85)	1.10 (0.64 - 1.90)	1.57 (0.91 - 2.74)	0.11	1.00	0.86 (0.47 - 1.55)	0.73 (0.39 - 1.38)	0.58 (0.30 - 1.14)	0.098
n-6 PUFA/n-3 PUFA	1.00	0.73 (0.42 - 1.30)	1.28 (0.78 - 2.10)	1.45 (0.88 - 2.38)	0.042	1.00	1.16 (0.63 - 2.14)	0.70 (0.36 - 1.37)	1.03 (0.55 - 1.93)	0.71

Soy foods	1.00	1.32 (0.81 - 2.16)	0.88 (0.51 - 1.50)	1.12 (0.66 - 1.90)	0.93	1.00	1.00 (0.55 - 1.84)	0.77 (0.40 - 1.48)	0.96 (0.51 - 1.81)	0.71
Meat	1.00	0.97 (0.60 - 1.57)	0.56 (0.33 - 0.97)	0.85 (0.51 - 1.40)	0.24	1.00	1.05 (0.58 - 1.89)	1.01 (0.54 - 1.88)	0.52 (0.26 - 1.05)	0.093
Fish	1.00	0.92 (0.56 - 1.49)	0.79 (0.48 - 1.30)	0.89 (0.53 - 1.51)	0.53	1.00	0.55 (0.28 - 1.07)	0.77 (0.41 - 1.45)	1.29 (0.71 - 2.36)	0.33
Green-yellow vegetables	1.00	1.14 (0.70 - 1.86)	0.88 (0.52 - 1.49)	1.11 (0.65 - 1.90)	0.95	1.00	0.76 (0.42 - 1.38)	0.70 (0.38 - 1.28)	0.58 (0.30 - 1.12)	0.095
Other vegetables	1.00	1.47 (0.91 - 2.40)	0.96 (0.56 - 1.65)	0.92 (0.54 - 1.59)	0.41	1.00	0.81 (0.45 - 1.48)	0.92 (0.50 - 1.68)	0.72 (0.37 - 1.37)	0.40
Fruit	1.00	1.66 (1.01 - 2.72)	1.46 (0.86 - 2.49)	1.04 (0.58 - 1.86)	0.99	1.00	1.00 (0.53 - 1.88)	0.74 (0.37 - 1.49)	1.47 (0.79 - 2.73)	0.33

* : adjusted for age, year of first visit to the hospital, season of first visit, reason for the visit, family history of colorectal cancer, body mass index, exercise, alcohol drinking, smoking, multivitamin use, and energy intake.

† : saturated fatty acids

‡ : monounsaturated fatty acids

§ : polyunsaturated fatty acids

95% confidence intervals in parentheses

## DISCUSSION

In this case-control study, we found a decreased risk of colon cancer with increasing intakes of calcium and insoluble dietary fiber, while a higher consumption of carotene and meat was associated with a reduced risk of rectal cancer. Carbohydrate intake was linked to the risk of rectal cancer, particularly in women, while fat consumption was inversely correlated with the risk of colon and rectal cancers in women.

People take much less meat and more cereals in Asian countries than in the United States and Canada (http://faostat.fao.org/faostat/), which may account for the lower colon-to-rectal ratios in Asia. Further investigations, however, are needed because the differences in risk for the consumption of meat and carbohydrate between colon and rectal cancers have not been fully supported by previous investigations.

Calcium intake has been related to a decreased risk of colorectal cancer in prospective studies.^16^^-^^18^ In addition, randomized controlled trials showed that calcium supplementation prevents recurrence of colorectal adenomas, precursors of cancers.^19^ The present study provides further support for role of calcium in the prevention of colorectal cancer. Some investigations demonstrated a greater risk reduction for cancer of the colon than that of the rectum as in our case.^16^^,^^18^

An inverse association of dietary fiber and colon cancer risk was here detected specifically for insoluble dietary fiber. Many epidemiologic studies have not substantiated a protective association between dietary fiber and colorectal cancer,^20^^-^^22^ although a recent large prospective study in Europe showed a decreased risk of colorectal cancer associated with dietary fiber intake.^23^

An earlier investigation reporting protective effects of dietary fiber against colorectal cancer did not find a substantial difference in risk between soluble and insoluble fibers.^24^ Whereas adsorption of carcinogens to insoluble dietary fiber in the intestinal tract is one of the mechanisms by which dietary fiber is believed to protect against colorectal cancer,^25^ the roles of different types of fiber should be further elucidated. Our finding that the association of dietary fiber was mainly with colon rather than rectal cancer is consistent with the finding of the European study mentioned above^23^ and might be expected because the rectum is empty most of the time, reducing the putative protective effects of dietary fiber.^23^

The intake of carotene was negatively associated with the risk of rectal cancer, particularly in female subjects. Women who consumed much green-yellow vegetables tended to show a lower risk of rectal cancer. A risk-reducing effect of dietary carotene was suggested for rectal cancer in some earlier case-control studies.^4^^,^^26^^,^^27^ The association, however, should be further examined in cohort studies because several large prospective studies have recently pointed to no preventive effects of fruit and vegetables, which are rich in carotene.^28^^-^^30^

Although red meat consumption has been linked to the risk of colorectal cancer,^31^^-^^33^ we have failed to find any positive association of meat and in fact observed a relation to a somewhat decreased risk for rectal cancer. In Japan, red meat accounts for a smaller proportion of all meat consumption than in Western countries (http://faostat.fao.org/faostat/) and colorectal cancer risk may be reduced or unaltered by non-red meat,^32^^,^^34^ so this might explain the lack of any deleterious effect of meat in our study. Our finding is consistent with the results of a case-control study in China, which reported an increased risk of rectal cancer associated with reduced consumption of meat.^5^ Another case-control study conducted in Japan also reported that meat consumption was inversely correlated with the risk of rectal cancer.^6^

A high correlation between national per capita intake of fat and national rates of colon cancer has led to the hypothesis that consumption of fat increases risk of colon cancer. In general, however, neither case-control nor cohort studies have provided unequivocal support for this hypothesis.^35^ In our study, intakes of fat, cholesterol, and monounsaturated fatty acids were inversely correlated with the risk of female colon and/or rectal cancer whereas a higher intake of carbohydrate was associated with rectal cancer risk, especially in women.

Some^36^^,^^37^ but not all^38^^,^^39^ case-control and cohort studies have suggested that higher intake of carbohydrate may increase colorectal cancer risk and this has been discussed in relation to insulin resistance. If this is the case, higher intake of fat and protein relative to carbohydrate may seemingly be linked to a decreased risk. As we suggested for colon cancer, Franceschi et al^40^ found a decreased risk associated with a higher intake of protein.

Several significant associations between dietary variables and colon or rectal cancer risk appeared in women but not in men. This may partly be attributable to the difference in intake levels of nutrients or food groups between the sexes. For example, men took more carbohydrate (median of the estimated intake in controls: 242.6 g/day in men versus 207.2 g/day in women) but less green-yellow vegetables (median in controls: 49.5 g/day versus 69.2 g/day) from our present data (values are adjusted to the mean energy intake of 1,710 and 1,493 kcal/day for men and women, respectively). On the other hand, variation by sex may be due to random fluctuation because the numbers of cases by site of cancer and sex were relatively small.

Dietary risk factors could be directly compared between colon and rectal cancers in the present study because the procedures for identification of cases and data collection were exactly the same and the control group was common for the two sites of cancer.

Some methodological limitations, however, need consideration. First, because this was a hospital-based case-control study, the source population from which cases arise may differ from that for controls. To take this into consideration, we adjusted for the reason for the first visit to ACCH and the season. Second, as with other case-control studies, this study may suffer from recall bias. Although the questionnaires were completed before the diagnosis in ACCH, some case patients referred to the hospital might have known the diagnosis. It is unlikely, however, that the recall bias affected the findings differentially between colon and rectal cancers. Third, because we examined many nutrients and food groups in relation to the risk of colon and rectal cancers, multiple comparisons may be another issue. Some findings might have appeared by chance. The difference in dietary risk factors between colon and rectal cancers found in the present study, therefore, warrant confirmation in further investigations. The increase in incidence of colorectal cancer over time^1^ may mean that most Japanese have changed their lifestyles, including their dietary consumption, so that detection of dietary risk or protective factors in case-control or cohort studies within the Japanese population faces particular problems. Finally, the limitations of the questionnaire may have prevented us from considering some potential confounding factors. For example, no information was available on non-steroidal anti-inflammatory drugs (NSAIDs), which may exert protective effects against colorectal cancer^41^ and may confound associations between diet and the risk of cancer.

In conclusion, dietary preventive factors appear to considerably differ between colon and rectal cancers: calcium and insoluble dietary fiber may protect against colon cancer while carotene and meat may be more effective for rectal cancer. Carbohydrate intake was positively correlated with the risk of rectal cancer, especially in women.
